# Preparation of α-mannoside hydrogel and electrical detection of saccharide-protein interactions using the smart gel-modified gate field effect transistor

**DOI:** 10.1186/1556-276X-7-108

**Published:** 2012-02-07

**Authors:** Yasuhiro Maeda, Akira Matsumoto, Yoshiko Miura, Yuji Miyahara

**Affiliations:** 1 Institute of Biomaterials & Bioengineering, Tokyo Medical and Dental University, 2-3-10 Kanda-Surugadai, Chiyoda-ku, Tokyo, 101-0062, Japan; 2 Department of Chemical Engineering, Kyushu University, 744 Motooka, Nishi-ku, Fukuoka, 819-0395, Japan

**Keywords:** Saccharide-protein interaction, field effect transistor, potentiometry, soft interface, concanavalin A, stimuli-responsive gel, molecular recognition.

## Abstract

The purpose of this study was to detect saccharide-protein interaction capitalizing on the gel-modified field effect transistor [FET]. A lectin-sensitive polymer gel that undergoes volume changes in response to the formation of molecular complex between 'pendant' carbohydrate and a 'target' lectin concanavalin A [Con A] was synthesized. It was revealed that direction and magnitude of the gel response (swelling or deswelling) could be readily designed depending on composition and network density of the gel. The Con A-sensitive polymer gel has shown the ability to transduce the detection of saccharide-protein interactions into electrical signals for FET.

**PACS: **87.85.jf, bio-based materials

## Introduction

Field effect transistor [FET]-based bio-sensing, so-called bio-FET, is an emerging class of label-free sensing format applicable to a range of biological targets, which can be readily miniaturized and integrated by virtue of advanced semi-conductor processing technology. In principle of bio-FET, any 'programmed' charge density changes on the gate surface can be detected as a mode of the modified channel characteristics of the FET in synchronization with electro-static interactions between these charges and the thin insulator-segregated silicon electrons. The bio-FET, however, while offering a number of promising applications, is susceptible to the charge-screening effect caused by counter ions [1,2]. As a result, the technique yields a short detectable length limit (from the gate surface), which corresponds to the thickness of the electrical double layer or the Debye length of up to a few nano-meters at most with minimized ionic strength of the environment [3]. This leads to an upper limit of the molecular weight for which quantitative charge detection can be feasibly performed [1,3,4].

To address this, we have recently proposed exploitation of a stimuli-responsive polymer gel as a signal-transducing material bridging between the target and the gate insulator [5,6]. Stimuli-responsive polymer gels or 'smart gels' are a unique class of material capable of undergoing marked changes in their physicochemical properties in response to a series of specific stimuli. In particular, those sensitive to chemical stimuli, i.e., concentration fluctuations of specific molecules, are of significant interest due to their potential impact on clinical applications including bio-materials, drug delivery systems and actuators. In the gate-introduced configuration, smart gels, upon applied stimuli, can evoke an abrupt volume change termed as 'volume phase transition' causing other physical parameter changes such as thickness, charge density and permittivity. As a key feature, these physicochemical changes commencing at the gel/outer aqueous media interface can geometrically propagate across a macroscopic thickness of the gel layer and are, thus, able to transport the signal beyond the 'barrier' of the Debye length.

**Figure 1:**
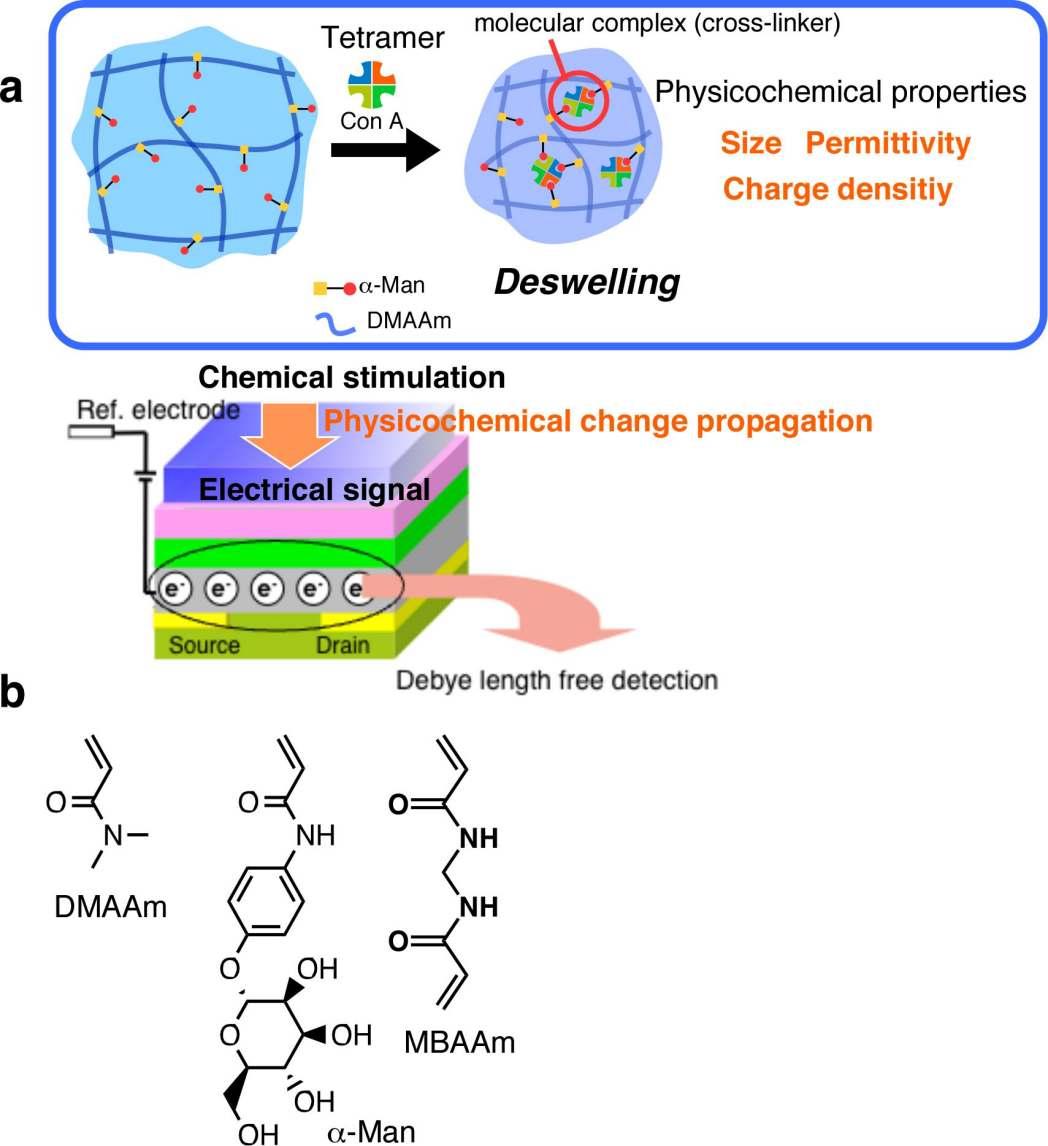
**Conceptual scheme for a lectin-responsive gel-mediated signal transduction enabling a 'Debye-length-free' FET-based Con A detection**. (**a**) Mechanism of detection of saccharide-protein interaction capitalizing on the gel-modified FET. (**b**) Chemical structures of the components of lectin-responsive gel.

This paper focuses on detection of saccharide-protein interaction capitalizing on the gel-modified FET (Figure 1a). Saccharides on the cell surfaces play important roles in the life systems including cell-cell communication, immune response, pathogen invasion and cancer metastasis [7]. Because saccharide-protein interactions are central to these phenomena, a better understanding of saccharide functions would greatly aid in the elucidation of life and, hence, diagnosis and detection of diseases and pathogens [8]. First, we will describe the preparation of a lectin-sensitive polymer gel that undergoes volume changes in response to the formation of molecular complex between carbohydrate and a lectin concanavalin A [Con A] (Figure 1). It was revealed that the direction and magnitude of the gel response (swelling or deswelling) could be readily designed depending on composition and network density of the gel. In the latter part, some preliminary detection of saccharide-protein interactions will also be demonstrated.

## Experimental details

### Materials

A monomer bearing a pendant mannose (*p*-acrylamidophenyl-α-D-mannoside [α-man]) was prepared via procedures reported previously [9]. *N, N*-dimethylacrylamide [DMAAm] (Wako Pure Chemical Industries, Ltd., 3-1-2, Doshomachi, Chuo-Ku, Osaka, 540-8605, Japan) was purified under reduced pressure (13 mmHg) in a nitrogen atmosphere before use. *N, N*'-methylenebisacrylamide [MBAAm], 2,2'-Azobis(2-methylpropionamidine) dihydrochloride (V50), acryloyl chloride, dimethyl sulfoxide [DMSO], anhydrous ethanol, manganese (II) chloride tetrahydrate, calcium chloride (Wako Pure Chemical Industries, Ltd.), 1-[4-(2'-hydroxyethoxy)-phenyl]-2'-hidroxy-2"-methyl-1-propane-1-one (Irgacure2959) (Ciba Specialty Chemicals, Inc., Klybeckstrasse, 141 CH-4002 Basel, Switzerland), Con A (lectin from *Canavalia ensiformis*) (J-Oil Mills, 1-11-1, Marunouchi, Chiyoda-Ku, Tokyo, 100-6226, Japan), 4-(2-hydroxyethyl)-1-piperazineethanesulfonic acid [HEPES] and 11-amino-1-undecanethiol, hydrochloride [AUT] (Dojindo Molecular Technologies, Inc., Kumamoto Techno Research Park, 2025-5 Tabaru, Mashikimachi, Kamimashiki gun, Kumamoto 861-2202, Japan) were all used without further purification. Deionized ultra-pure Milli-Q water (Millipore, 290 Concord Road, Billerica, MA, 01821, USA) (18.2 MΩ cm^-1^) was used throughout the experiments. Hydrofluoric acid [HF] (23%) was prepared by diluting 46% HF (Morita Chemical Industries, Co., Ltd., 4-1-3, Kyutaromachi, Chuo-Ku, Osaka, 541-0056, Japan) with water.

### Preparation of buffers

HEPES buffers (20 mM) were prepared using recipes from buffer calculator [10,11] developed by R. Beynon at the University of Liverpool, UK. The buffers were titrated to a specific pH with KOH solution, and the overall ionic strengths were fixed with KCl.

### Preparation of α-man hydrogel

DMAAm, α-Man, MBAAm (as a crosslinker) and V50 (as an initiator) were dissolved in 50:50 (*v/v*) DMSO/water-mixed solvent as shown in Table 1. The total monomer concentration (DMAAm and α-Man) was either 2 or 4 M. The radical co-polymerization was carried out in a glass capillary (internal diameter [ID] = 0.93 mm; d_t_) (Drummond Scientific Company, 500 Parkway, Box 700, Broomall, PA, 19008, USA), of which, both ends were sealed with an epoxy resin at 50°C for 24 h. After the reaction, the glass capillary was dissolved by immersing it in a 23% HF solution for 6 h. The obtained α-man gel was immersed in water and dialyzed in HEPES buffer (pH = 6.9, 25°C, *I *= 0.3 M) for 48 h to remove any remaining unreacted components. The purified gel was cut into pieces of 1-cm length, transferred to and equilibrated in a HEPES buffer (pH = 6.9, 4°C, *I *= 0.3 M) containing CaCl_2 _(1 mM) and MnCl_2 _(1 mM).

**Table 1:** Composition of functional monomer in pregel solution

Hydrogel	DMAAm (g)	α-man (M)	Content of α-man (mol%)	MBAAm (mM)	V50 (mM)
2D	2.00	0.00		20.00	50.00
2DS3	1.94	0.06	3.00	20.00	50.00
2DS20	1.60	0.40	20.00	20.00	50.00
2DS80	0.40	1.60	80.00	20.00	50.00
4DS3	3.88	0.12	3.00	40.00	50.00
4DS20	3.20	0.80	20.00	40.00	50.00

DMAAm, *N, N*-dimethylacrylamide; MBAAm, *N, N*'-methylenebisacrylamide.

### Swelling ratio

After equilibration at 4°C, the surface water of a piece of the gel was removed by gentle blotting with a laboratory tissue and was weighted (*W*_0_). The gel was transferred to a HEPES buffer containing Con A (10 μM) in the presence of both CaCl_2 _(1 mM) and MnCl_2 _(1 mM). The gel weight at given duration (*W*) was measured and the swelling ratio of the gel was defined as in Equation 1:

(1) $$\text{Swelling ratio }\left[\%\right]=\left\{\left(W-W_{0}\right)/W_{0}\right\}\times 100$$

### Modification of gold electrode with α-man gel

A gold electrode (5 × 5 mm^2^) was fabricated by the sputter deposition of an adhesion layer of titanium (10 nm) and then of a gold layer (90 nm, 99.99% purity) on a silicon substrate (Ferrotec silicon; Ferrotec Corporation, 1-4-14 Kyobashi, Chuo-ku, Tokyo, 104-0031, Japan). The gold electrode was cleaned before use with acetone, ethanol, water and, finally, piranha solution (H_2_O_2_/H_2_SO_4 _= 25/75 *v/v*) (extreme caution must be exercised when using piranha etch; an explosion-proof hood should be used). The surface was rinsed thoroughly with water and ethanol, and was dried with nitrogen gas. An AUT self-assembled mono-layer [AUT SAM] was formed on a clean gold electrode by immersing the electrode in a 2-mM AUT ethanol solution under nitrogen atmosphere at room temperature for 24 h. After repeated rinses and sonication in pure ethanol for 5 min, the electrode was dried with nitrogen gas. The AUT SAM, thus, obtained was then treated with acryloyl chloride under nitrogen atmosphere at room temperature for 6 h to modify the SAM terminus amino groups with acryloyl groups. After the reaction, the electrode was washed by sonication in ethanol for 5 min and rinsed with water. The electrode was covered with a teflon tape with a round hole (thickness = 50 μm, *φ *= 6 mm), in a way, exposing the central gold surface to the air.

A pregel solution was prepared by dissolving DMAAm, α-man, MBAAm as a crosslinker and Irgacure2959 as an initiator in DMSO solvent as shown in Table 2. α-man gel micro-film was synthesized by pipetting 1- μl droplets of the pregel solution onto the gold surface, which was then covered with a cover slip and placed under UV light (500 mW/cm^2^, Aicure UJ20; Panasonic Electric Works Co., Ltd., 1048 Kadoma, Kadoma-Shi, Osaka, 571-8686, Japan) to allow radical co-polymerization.

**Table 2:** Composition of functional monomer in pregel solution for photo-polymerization

Hydrogel	DMAAm (g)	α-man (M)	Content of α-man (mol%)	MBAAm (mM)	Irgacure2959 (mM)
4DS20	3.20	0.80	20.00	40.00	50.00
4DS50	2.00	2.00	50.00	40.00	50.00

DMAAm, *N, N*-dimethylacrylamide; MBAAm, *N, N*'-methylenebisacrylamide.

### Fabrication of extended gate-FET bio-sensor

A reaction chamber (200 μL volume) was made on the electrode. A glass tube (ID = 5 mm) was immobilized on the gold surface with a thermosetting-insulating epoxy resin (XA-1295/HQ-1) (Pelnox, Ltd., 8-7 Bodai, Kanagawa, 259-1302, Japan) by heating at 60°C for 12 h. The rest of the substrate, including bonding wires, had to be completely protected against water penetration from the reaction solution so the periphery of the chamber was completely covered with the epoxy resin. HEPES buffer (pH = 6.9, 4°C, *I *= 0.3 M) containing CaCl_2 _(1 mM) and MnCl_2 _(1 mM) was pipetted into the reaction chamber. The source and drain of a commercially available FET (N-Channel Depletion-Mode MOSFET, LND150) (Supertex Inc., 1235 Bordeaux Drive, Sunnyvale, CA, 94089, USA) and Ag/AgCl reference electrode were connected to a real-time FET analyzer (Optogenesis, 2-1-8 Kenpuku, Honjo-Shi, Saitama, 367-0044, Japan), and the gate of the FET was connected to the wire, which was connected to separated α-man gel-modified gold electrode.

### Detection of Con A using α-man gel-modified FET

The α-man gel-modified gold electrode was equilibrated in HEPES buffer (pH = 6.9, 4°C, *I *= 0.3 M) containing CaCl_2 _(1 mM) and MnCl_2 _(1 mM) prior to use. Real-time change in the gate potential was monitored and recorded at source current (*I*_S_) of 1,800 μA and gate voltage (*V*_G_) of 0 V (versus Ag/AgCl) when fixing source-drain voltage (*V*_D_) at 1 V. Various concentrations of Con A solution were prepared in the HEPES buffer. Con A detection was carried out by adding 5 μM Con A into the reaction chamber and incubated at 25°C.

## Results and discussion

### Synthesis of α-man gels

**Figure 2:**
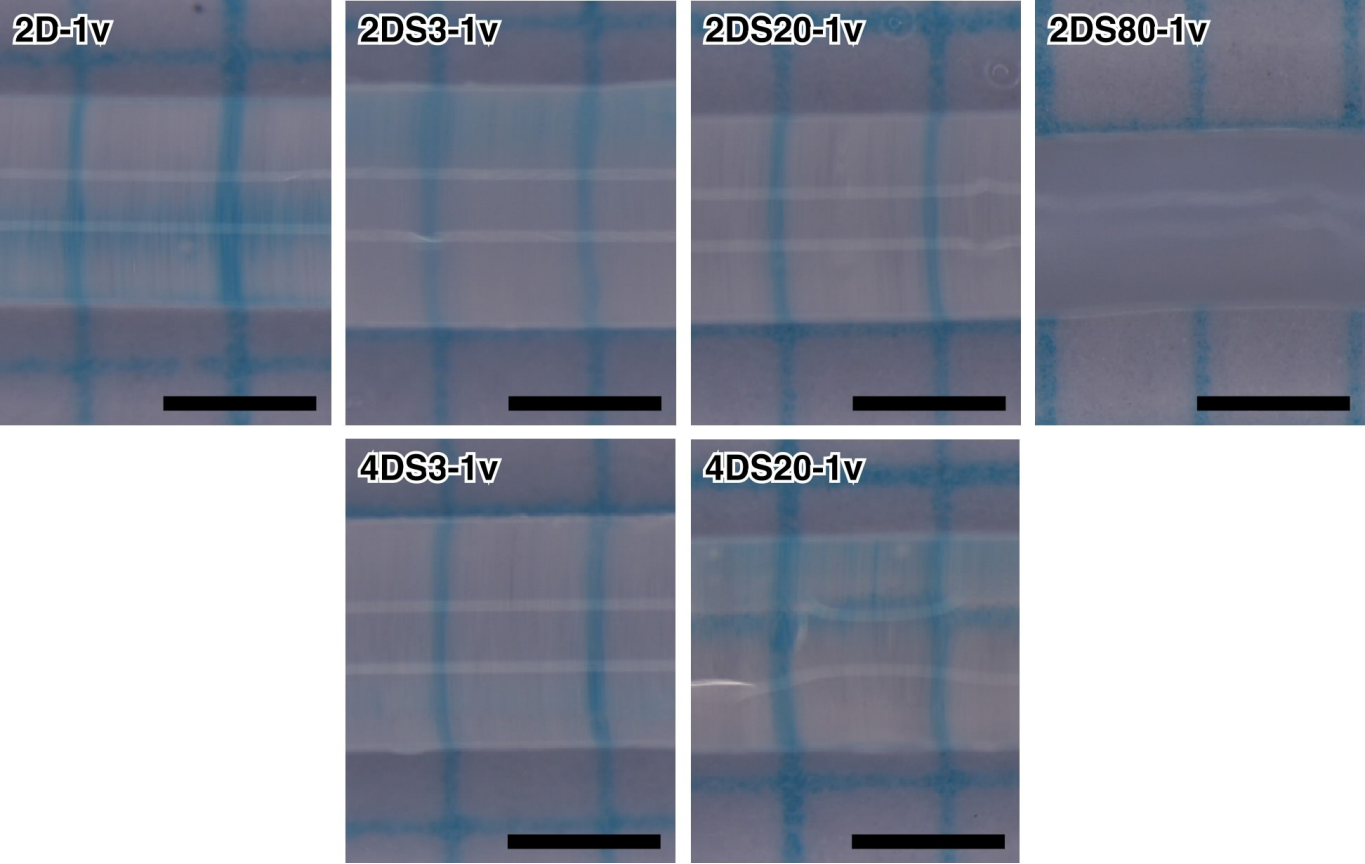
**Optical microscope images of α-man gels with various network densities and contents of α-man**. All are equilibrated at 4°C (scale bar: 1 mm).

Several different types of α-man gels were synthesized by radical co-polymerization in shape of capillary (Table 1). As shown in Figure 2, molar ratio of the α-man was found to have significant impact on the diameter of the obtained gels (d/d_t_). When the gels were prepared from 2 M total monomer concentration (2D, 2DS3, 2DS20 and 2DS80), a maximal equilibrium state diameter was found at 3 mol% of the α-man content. It is interesting to note that, at this critical value of the α-man content, the gel yielded in slightly white and opaque color which was not observed for those with 0 or 20 mol% α-man (2D and 2DS20), whereas a gel with 80 mol% α-man (2DS80) showed more profoundly white and opaque color. Transparency of a gel is an indicator of its own structural uniformity in the scale of visible light wavelength (several hundred nano-meters). Namely, with increased heterogeneity in this scale of topological structure of the gel, a fraction of scattered visible light (through the gel) increases, making the gel appear white and opaque. Therefore, observations in Figure 2 indicate that the composition of the α-man significantly affects the nano-structure of the resulting gel. We postulate that a strong hydrophobicity of the α-man monomer plays a role to induce micro- or nano-phase separation during the polymerization. The magnitude is dependent on both the concentration of itself as well as the polarity of the reaction solvent used. Preparation and characterization of such nano- and micro-structured hydrogels have been reported previously [12-15]. Advantageously enough, it has been demonstrated that such heterogeneous gels are able to swell (hydrate) to a greater extent compared to homogeneous gels. This is also consistent with the results obtained in Figure 2. As will be discussed later, when introduced onto the gate surface of the FET, the swelling (hydration) degree of the gel is a determinant for both the signal direction and amplitude. This means that controlled nano-structure of the gel provides a means to modulate electrical signals that we actually observe.

### Con A-responsive behavior of α-man gels

Lectin binding to a single saccharide ligand is typically a low-affinity interaction. However, the multi-valent nature of lectin-saccharide interactions allows many low-affinity binding events to occur, resulting in high overall avidity [16]. Multimeric lectins can cross-link multi-valent carbohydrate ligands, and they selectively cross-link with a single species of glycoprotein to form uniform lectin-carbohydrate lattices [17]. Since tetrameric Con A can bind with four independent carbohydrates at once, one can anticipate that Con A-α-man complexation would result in inter- or intra-chain cross-linkages and, thus, deswelling of the gel (Figure 1a).

**Figure 3:**
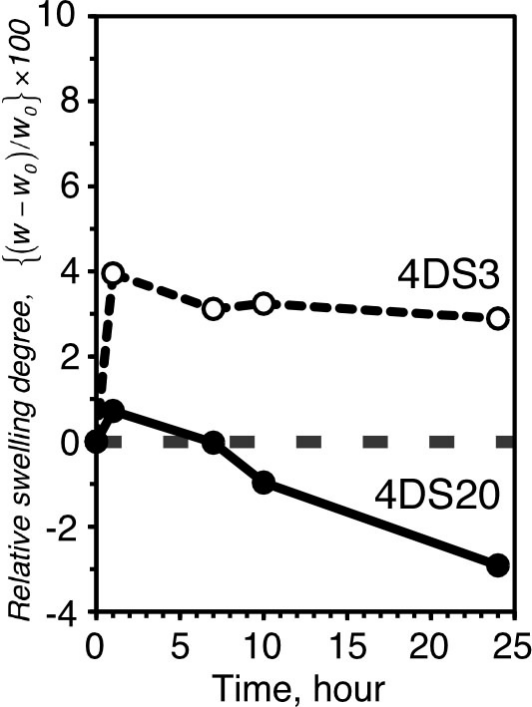
**Time course swelling ratio changes of α-man gels in response to 10 μM Con A**. 4DS3, Empty circles with round broken lines; 4DS20, filled circles with straight line.

**Figure 4:**
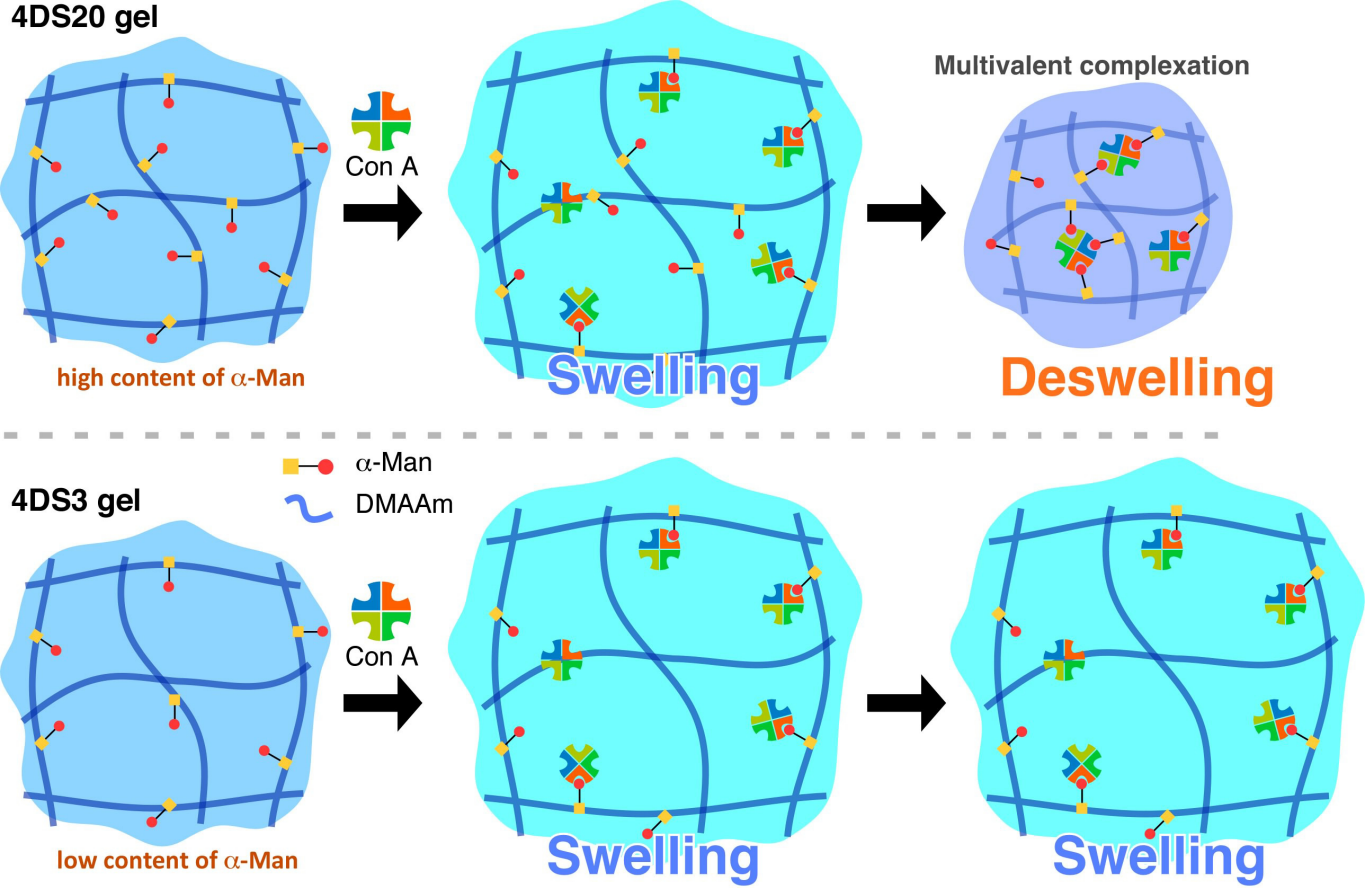
**Mechanisms of the two distinct Con A-responsive behavior of α-man gel**.

Figure 3 shows time courses of the swelling degree of the gels with the addition of Con A. In both cases, the gels swelled immediately after the addition of Con A; the extent of which is more prominent for 4DS3. At early stage of the α-man-Con A binding, it may occur in a 1 to 1 fashion rather than multi-valent complexation (Figure 4). Then a weakly (anionically) charged Con A should contribute to increase osmotic pressure of the gel, which would help the gel to swell rather than shrink (Figures 3 and 4). At later stage, as the thermal motion of the polymer chain matures, chances may increase for the multi-valent α-man-Con A complexation, which makes the gel deswell as observed in 4DS20 (Figures 3 and 4). 4DS3 gel bearing much less amount of α-man compared to 4DS20 did not show any significant deswelling even after 24 h. This can be explained by an insufficient number of α-man units available for multimeric recognition in the network, preventing the inter- or intra-chain cross-linkages. These results demonstrate feasibility to control the direction (swell or deswell) of the gel response simply by modulating the content of α-man.

### Con A detection using α-man gel-modified FET

As proposed in our previous work [6], the expression of threshold voltage [*V*_T_] of the gel-modified FET is given by Equations 2 and 3:

(2) $$V_{\text{T}}=E_{\text{ref}}-\psi_{\text{0}}+\chi^{\text{sol}}-\frac{\Phi_{\text{Si}}}{q}-\frac{Q_{\text{OX}}+Q_{\text{SS}}+Q_{\text{B}}+Q_{\text{gel}}}{C_{\text{Com}}}+2\Phi_{\text{f}}$$

with

(3) $$C_{\text{Com}}=\frac{C_{\text{OX}}\times C_{\text{gel}}}{C_{\text{OX}}+C_{\text{gel}}}=\frac{C_{\text{OX}}}{1+\frac{C_{\text{OX}}}{C_{\text{gel}}}}$$

where *E*_ref _is the constant potential of the reference electrode, *ψ*_0 _is the potential drop in the electrolyte at the insulator-electrolyte interface, *χ*^sol ^is the surface dipole potential of the solution, $\frac{\Phi_{\text{si}}}{q}$ is the silicon electron work function, the fifth term is due to accumulated charge in the oxide(*Q*_OX_) at the oxide-silicon interface (*Q*_SS_) and the depletion charge in the silicon (*Q*_B_), Φ_*f *_is the potential difference between the Fermi levels of doped and intrinsic silicon, *Q*_gel _is the charge in the gel layer and *C*_Com _is the combined capacitance of the gate oxide (*C*_OX_) and the α-man gel layer (*C*_gel_). All other variables than *Q*_gel _and *C*_gel _are regarded constant throughout the chemical stimulation to the gel. On the basis of the operation function of FET (Equation 2), an increased anionic density on the gate surface gives rise to a positive directional shift of *V*_T_, whereas a decreased anionic density gives a negative directional shift of *V*_T_. Likewise, an increase in the gel permittivity on the gate surface leads to a negative direction shift of *V*_T _and *vice versa*.

**Figure 5:**
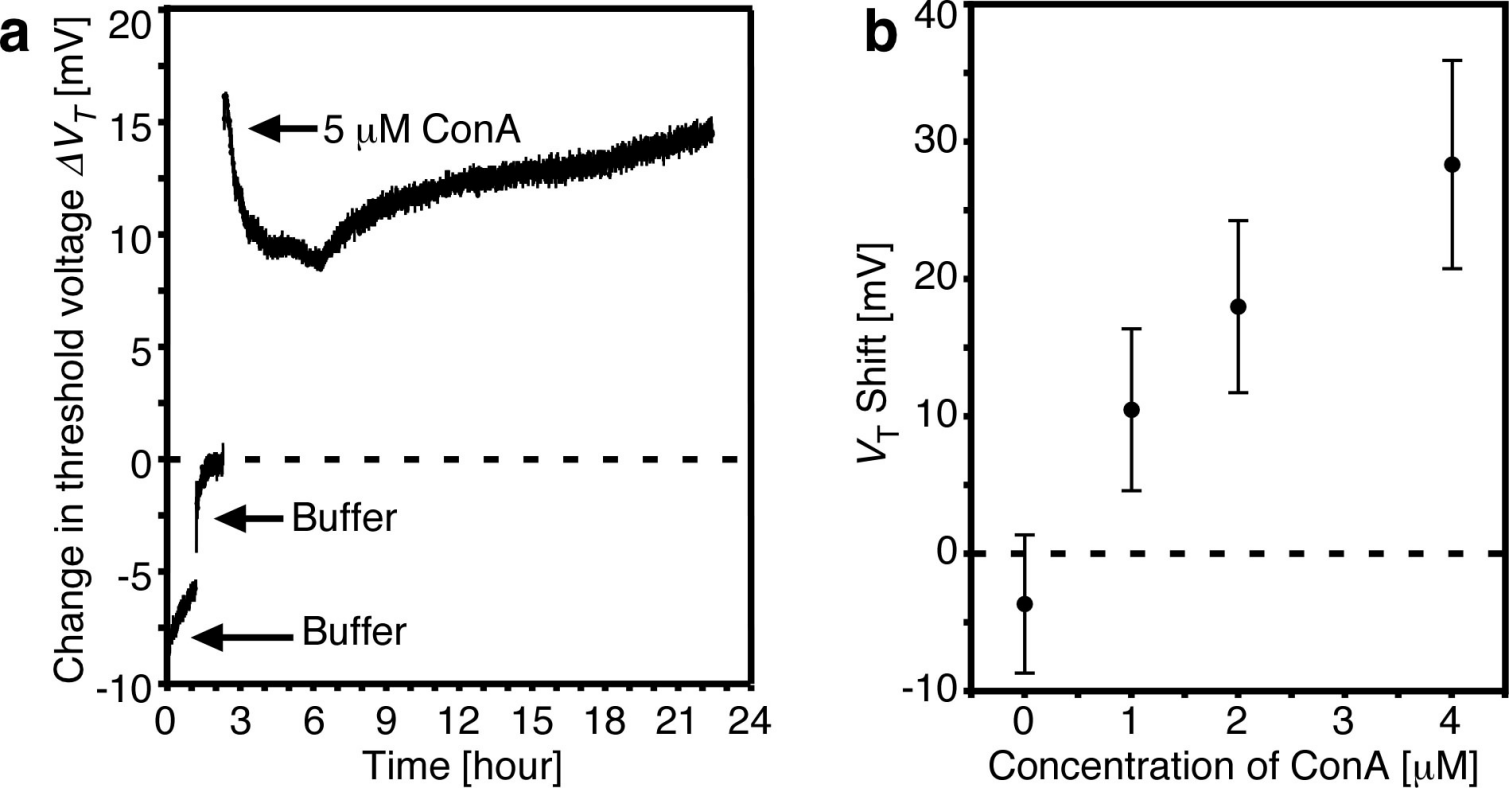
**Detection of Con A using α-man gel-modified FET**. (**a**) Time course changes of threshold voltage [*V*_T_] of 4DS20-modified FET for additions of buffer with and without 5 μM Con A investigated at 25°C (pH = 6.9, *I *= 0.3 M). (**b**) *V*_T _shifts as a function of Con A concentration on the 4DS50-modified FET investigated at 25°C (pH = 6.9, *I *= 0.3 M) (*n *= 3).

Figure 5a shows time course response of *V*_T _of 4DS20-modified FET upon addition of 5 μM Con A. *V*_T _undergoes a three-stage response: the first was an immediate and abrupt increase (stage 1) and the second response was a gradual decrease (stage 2) followed by slow re-increase (stage 3). The shrinking and swelling processes of the polymer gels take place in order of (1) diffusion of the solute molecules in the polymer network, (2) relaxation of the polymer chains due to (de) solvation and (3) diffusion of the polymer chains into (or out of) the solvent [18]. From the directions of the *V*_T _shifts observed in Figure 5a and in light of the above discussion, the stage 1 response can be attributed to the charge effect due to both diffusion of Con A (pI = ca.5-7) [19-23] into the hydrogel and the complexation with α-man (in a 1:1 fashion) corresponding to step 1 of the gel response. On the other hand, the secondary and third changes can be attributed to the change in permittivity of the gel. It should be noted that the volume change of a hydrogel is practically equivalent to the change in water content. That is to say, as the gel deswells over time (stages 2 and 3), the fraction of high permittivity water decreases, decreasing the capacitance factor of the gel/gate interface. This safely explains the observed recovery of *V*_T _at later stages of the response. The *V*_T _shifts of 4DS50-modified FET as a function of Con A concentration were shown in Figure 5b. A data point at 1 h after the addition of the solution was defined as the equilibrium *V*_T _shift for buffer without Con A, and a data point at the end of the second stage was defined as the equilibrium *V*_T _shift for Con A-containing buffer. When the Con A-containing buffer was added to the gel-modified FET, positive shifts of the *V*_T _were observed and increased as the Con A concentration increased. This result shows the potential of the α-man gel-modified FET for detecting saccharide-protein interaction. These results show that the gel-modified FET is not only able to electrically detect weakly charged proteins of large molecular weights on the basis of protein-saccharide interactions but also reveal kinetics of the signal transduction.

## Conclusions

In this study, lectin-sensitive polymer gels showing volume changes in response to the formation of molecular complex between pendant carbohydrate and target lectin Con A were prepared. The direction and magnitude of the gel response (swelling or deswelling) could be readily designed depending on composition and network density of the gel. The α-man gel-modified FETs not only showed the ability to electrically detect weakly charged proteins of large molecular weights but also revealed kinetics of the signal transduction.

## Competing interests

The authors declare that they have no competing interests.

## Authors' contributions

YaM carried out the experiments, analyzed the data and drafted the manuscript. AM participated in the discussion of this research. YoM synthesized α-man monomer. YuM conceived of the study and participated in its design and coordination. All authors read and approved the final manuscript.
